# Prehabilitation in Frail Patients Undergoing Cancer Surgery: A Systematic Review and Meta-analysis

**DOI:** 10.1245/s10434-025-17589-y

**Published:** 2025-06-04

**Authors:** Zirong Bai, Cherry Koh, Michael Solomon, Rihan Shahab, Nicholas Hirst, Kate Alexander, Leani Souza Maximo Pereira, Ana Paula Drumond Lage, Daniel Steffens

**Affiliations:** 1 Surgical Outcomes Research Centre (SOuRCe), Royal Prince Alfred Hospital (RPAH), Camperdown, Sydney, NSW Australia; 2 Central Clinical School, Faculty of Medicine and Health, The University of Sydney, Sydney, NSW Australia; 3 Institute of Academic Surgery (IAS), Royal Prince Alfred Hospital, Sydney, NSW Australia; 4 Department of Geriatric Medicine, Royal Prince Alfred Hospital, Sydney, NSW Australia; 5 Postgraduate Program in Health Sciences, Faculdade Ciências Médicas de Minas Gerais (FCMMG), Belo Horizonte, MG Brazil

## Abstract

**Background:**

The evidence of prehabilitation in frail patients with cancer is lacking. This systematic review and meta-analysis aimed to determine the effectiveness of prehabilitation on postoperative complications, and hospital length of stay (LOS) in this population.

**Methods:**

A comprehensive search was performed in MEDLINE, Embase, Cochrane, CINAHL, AMED, and PsycINFO, encompassing all records from inception to December 2023. The outcomes of interest included postoperative complications and LOS. Risk of bias was assessed using the revised Cochrane risk of bias tool (RoB2) and GRADE was used to determine the quality of evidence. Relative risk (RR) or mean difference (MD) along with its 95% confidence interval (CI) were calculated by using random-effects meta-analysis.

**Results:**

Five randomised controlled trials (four trials in colorectal or colon cancer), including 466 patients (230 patients undergoing prehabilitation and 236 standard of care controls), were included. Prehabilitation reduced any postoperative complications (RR = 0.82; 95% CI = 0.71–0.95; four trials, *N* = 465), but no effect was observed for major postoperative complications (RR = 0.89: 95% CI = 0.71–1.11; two trials, *N* = 226) and LOS (MD = 0.3, 95% CI = −0.68 to 1.28; three trials, *N* = 349). A single trial (including 57 patients) investigated the effect of exercise-only on a range of postoperative complications, with no significant difference between groups observed.

**Conclusions:**

In our systematic review and meta-analysis, we found that prehabilitation significantly decreased the rate of any postoperative complications in frail patients with cancer undergoing surgery. The role of prehabilitation in improving major postoperative outcomes is unclear owing to the limited amount of evidence.

**Supplementary Information:**

The online version contains supplementary material available at 10.1245/s10434-025-17589-y.

Because of a rising population and anticipated increases in cancer incidence rates, there were an estimated 20 million new cancer cases and 9.7 million deaths worldwide in 2022.^1^ Surgery, providing significant long-term survival benefits, remains the primary curative treatment for these patients.^2^ However, it is important to recognize that surgery can also be a stressor for patients, potentially leading to a poorer overall prognosis.^3,4^ This includes consideration of major postoperative adverse events, such as infection, anastomotic leakage, and other complications, which may result in longer hospital stay, slower recovery, and poorer quality of life outcomes^5^

Moreover, a patient's preoperative conditions play a crucial role in determining their postoperative outcomes.^6^ Notably, frailty has been independently shown to predict postoperative complications, extended hospital stays, decreased quality of life, and increased morbidity.^7–9^ Frailty is an age-related state of cumulative and progressive decline in multiple physiological systems, leading to heightened vulnerability, which makes individuals particularly susceptible to significant functional declines when subjected to stressors.^10^ Patients with frailty are at a greater risk of postoperative complications, morbidity, mortality, and increased length of hospital stay.^11,12^ Hence, optimising frail patients’ health prior to cancer surgery may be crucial to improve their recovery.

Historically, rehabilitation has served as a foundational element in the recovery process of patients, emphasizing postoperative care. A poor preoperative health status can significantly increase the risk of postoperative complications and prolonged the length of hospital stay, and delay recovery.^13,14^ Emerging evidence indicates that prehabilitation, referred to as multidisciplinary preoperative optimization, encompassing physical, psychological, and nutritional support, represents a promising intervention for enhancing postoperative outcomes, particularly in patients with reduced preoperative fitness levels.^15–19^ This approach has the potential to mitigate postoperative complications, reduce hospital stays, lower readmission rates, and decrease overall healthcare costs.^18,20,21^

However, the effects of prehabilitation in frail patients with cancer remain inconclusive. Berkel et al.^16^ reported that prehabilitation significantly reduced postoperative complications in frail colorectal patients and suggested that it should be considered standard care for frail patients undergoing cancer surgery. Conversely, other studies found no difference between prehabilitation and usual care in frail patients with cancer.^4,22–24^ Although several systematic reviews have explored the effects of prehabilitation in frail patients undergoing cancer surgery, the results remain uncertain. Guo et al.^25^ conducted a systematic review to assess the effectiveness of prehabilitation on postoperative outcomes in frail patients with cancer undergoing elective surgery and found that prehabilitation positively impacted postoperative complications (relative risk [RR] = 0.83, 95% confidence interval [CI] = 0.73–0.94) and reduced the length of hospital stays (mean difference [MD] = −1.36, 95% CI = −2.38 to −0.35). However, the inclusion of both randomized controlled trials (RCTs) and historically controlled trials (HCTs) in these reviews may have introduced bias. Chang et al.^26^ revealed that prehabilitation has a significant effect in frail patients who underwent colorectal cancer surgery in postoperative complications (odds ratio [OR] = 0.51, 95% CI = 0.34–0.78) and length of hospital stay (standardised mean difference [SMD] = −0.34, 95% CI = −0.46 to 0.23), but this study only included three randomised controlled trials, including 283 patients. Previous reviews included meta-analyses of nonrandomised controlled trials or mixed interventions (e.g., preoperative geriatric liaison intervention, combined prehabilitation, and rehabilitation,), not in line with the current definition of prehabilitation (e.g, preoperative exercise, nutrition, and/or psychological interventions), which could lead to bias of the results.

Given the limited evidence from previous systematic reviews and the recent published randomised controlled trials, we conducted this systematic review and meta-analysis to determine the effectiveness of prehabilitation on postoperative complications and hospital stay length in frail patients undergoing cancer surgery.

## Methods

This review was conducted following the Cochrane handbook for interventions and reported according to the Preferred Reporting Items for Systematic Reviews and Meta-Analyses (PRISMA) guidelines.^27,28^ The protocol was registered at Open Science Framework.^29^

### Search Strategy

A comprehensive search was performed in the following databases: MEDLINE, Embase, The Cochrane Library, CINAHL, AMED, and PsycINFO, encompassing all records from inception to December 11, 2023 with no language limitation. The search strategy was formulated in collaboration with a librarian from The University of Sydney. The strategy was aligned with the methodological guidance provided by the Cochrane Handbook for Systematic Reviews of Interventions,^27^ specifically for randomised controlled trials. The search was supplemented by both forward and backward citation tracking to capture further relevant literature. Attempts were made to translate studies published in languages other than English to ensure comprehensive coverage. The complete search strategy is provided in the supplementary material (Supplementary Table S1).

### Inclusion and Exclusion Criteria

We included studies based on the following pre-determined criteria: (1) Adult patients (age 18 or older) who have been diagnosed with cancer and frailty (e.g., Clinical Frailty Scale); (2) Unimodal or multimodal interventions consisting of physical, psychological, and/or nutritional support delivered exclusively during the preoperative period; (3) Control group including standard care or minimal intervention; (4) Studies reporting postoperative measures of complication rates (e.g., presence of any postoperative complication, Clavien Dindo classification (CDC), comprehensive complication index (CCI), and/or length of hospital stay (LOS) (e.g., number of days patients spend in hospital after index surgery); and (5) Randomised controlled trials, quasi randomised controlled trials, or pilot randomised controlled trials were included in this review.

We excluded studies based on the following criteria: (1) Abstracts of conference proceedings/Commentaries/poster presentations/trial protocol/Clinical trials registration; (2) Ineligible subjects who are not frail patients with cancer; and (3) Unable to access the complete text or data despite attempts to contact the authors.

### Study Selection

The retrieved literature was imported into the COVIDENCE online software.^30^ Duplicate literature was removed. Two authors (ZB and NH) independently screened titles and abstracts and full-text articles. Studies not meeting the inclusion criteria were excluded. Study authors were contacted via email to seek full-text copies of studies or clarification of methods or results. Disagreements at each screening stage were resolved through discussion by a third reviewer (DS).

### Data Extraction

After study selection, two independent reviewers (ZB and NH) extracted information by using a predefined form. This form covers various aspects including study characteristics (i.e., publication year, country, and setting), description of intervention and comparator, adherence, and outcome measurements.

The outcomes of postoperative complications and LOS were recorded as number of events and mean (SD) values, respectively. When mean (SD) were not reported, an estimation was calculated from the median and range.^31^

### Risk of Bias (Quality) Assessment

The Risk of bias tool (RoB 2) from The Cochrane Collaboration was used to assess the included articles' risk of bias. Two independent reviewers (ZB and NH) undertook the risk of bias assessment for all included studies. Discrepancies were resolved by consensus, and any remaining issues were resolved by a third reviewer (DS). The guidelines provided by the current version of the Cochrane Handbook were used to assess each item’s risk of bias.

The quality of evidence for each outcome was evaluated using the Grades of Recommendation, Assessment, Development, and Evaluation (GRADE) approach.^32^ Study quality was graded as high (i.e., further research was very unlikely to change our confidence in the effect), moderate (i.e., further research was likely to have an important impact on our confidence in the effect and may change the estimate), low (i.e., further research was likely to have an important impact on our confidence in the effect and is likely to change the estimate), and very low (i.e., uncertain about the effect estimate).

### Strategy for Data Synthesis

We utilised the comprehensive meta-analysis software to conduct the analyses. For dichotomous outcomes, we calculated relative risk, and for continuous variables, we calculated mean difference (MD) along with its 95% confidence interval (CI). We applied a random-effects model to pool the data.

## Results

**Fig. 1:**
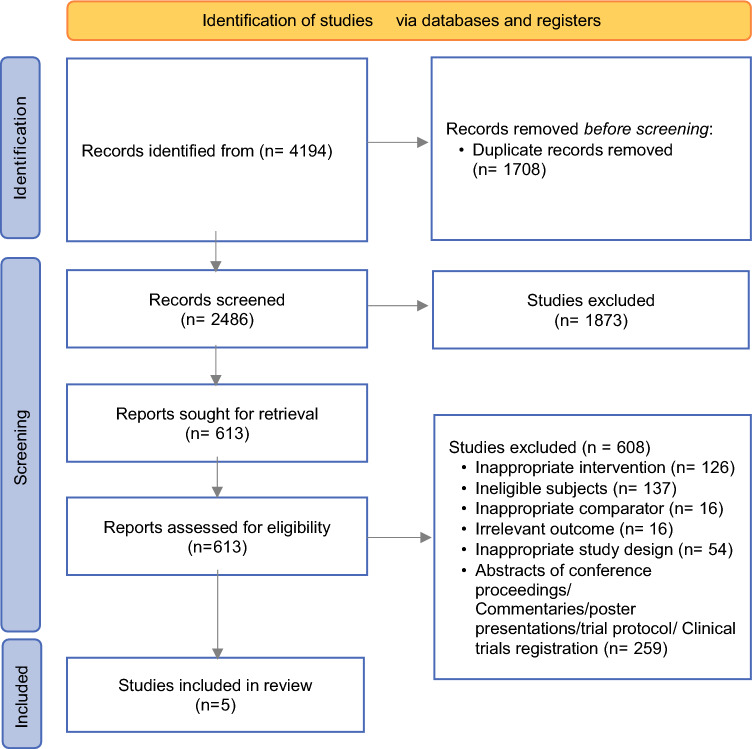
PRISMA flow diagram

The initial search resulted in 4194 articles; 1708 duplicate studies were excluded, of which 2486 remaining studies were screened for titles and abstracts, and 613 articles were included for full-text review. Of these, 608 studies did not fulfil eligibility criteria, resulting in a total of five full-text articles being finally included in this systematic review. Four articles were included in the meta-analysis.^16,22–24^ The PRISMA flow chart summarised the detailed selection process (Fig. 1).

### Characteristics of the Included Trials

The five included trials were published between 2018 and 2022 and conducted in four different countries: two in Canada, and one each in Holland, Australia, and Norway, including 230 patients undergoing prehabilitation and 236 standard of care controls.^16,22–24,33^ Surgical populations included colorectal, colon, intra-abdominal, or thoracic (colorectal, thoracic, hepatobiliary, or urologic), with the average age ranging from 73.0 to 80.2 years.^16,22–24,33^ The characteristics of the included trials are summarised in Table 1.

**Table 1:** Characteristics of included trials

Author, year	Characteristics	Intervention group		Control group		Reported outcomes
		Sample/mean age (SD)	Description	Sample/mean age (SD)	Description	
Berkel, 2022	Country: Holland Cancer type: Colorectal Frailty tool: Groningen frailty indicator Setting: Community	*N* = 28 Age = 74 (7)	Description: Exercise (aerobic, resistance training) Provider: Trained physical therapists Mode of delivery: Face-to-face Number of times: 3 sessions per week (9 sessions in total) Duration: 3 weeks Intensity: Moderate-to-high intensity Tailored: Yes Adherence: Not reported	*N* = 29 Age = 73 (6)	Description: Nutritional counselling and advice on smoking cessation. Duration: 2–3 weeks	Complications and length of hospital stay
Carli, 2020	Country: Canada Cancer type: Colon Frailty tool: Fried Frailty Index Setting: Home and hospital prehabilitation unit	*N* = 55 Age = 77.3 (7.6)	Description: Exercise (aerobic, resistance and stretching exercises); Nutrition (protein supplement); Psychological (personalised coping strategies) Provider: Kinesiologist, dietitian, psychology trained nurse Mode of delivery: Face-to-face Number of times: Supervised session (once per week), daily walking, elastic band training (three times per week), Relaxation (three times per week) Duration: 4 weeks Intensity: Moderate Tailored: Yes Adherence: Mean (SD): 80% (27%)	*N* = 55 Age = 80.2 (6.9)	Description: Exercise (aerobic, resistance and stretching exercises) Nutrition (protein supplement) Psychological (personalised coping) Provider: Kinesiologist, dietitian, psychology trained nurse Mode of delivery: Face-to-face Number of times: Once per week, daily walking, elastic band training (3 times/week), Relaxation (3 times/week) Duration: 4 weeks Intensity: Moderate Tailored: Yes Adherence: Mean (SD): 30% (33%)	Length of hospital stay
Furyk, 2021	Country: Australia Cancer type: Colorectal Frailty tool: Edmonton Frail Scale Setting: Hospital	*N* = 0 Age = not available	Description: Exercise (warm-up, strength, core/balance, aerobic exercise, cool down exercises), Nutrition (Dietary advice in accordance with Australian Dietary Guidelines) Provider: Qualified exercise physiologists Mode of delivery: face-to-face Number of times: 1-h sessions per week on non-consecutive days Duration: 4 weeks Intensity: Not reported Tailored: Yes Adherence: Not reported	*N* = 1 Age = 64	Description: Standard preoperative care involved an ad hoc, and as needed, program including continuation with medications and anxiety management if needed	Not available
Mclsaac, 2022	Country: Canada Cancer type: Intra-abdominal or thoracic (colorectal, thoracic, hepatobiliary, or urologic) Frailty tool: Clinical Frailty score Setting: Home-based	*N* = 94 Age = 74 (7)	Description: Exercise (strength training, aerobic exercise, flexibility); Nutrition (Foundational nutritional advice, motivation for healthy eating, and healthy eating and cooking tips) Provider: Not reported Mode of delivery: Face-to-face Number of times: 1 h sessions, done at least three times per week Duration: 4 weeks Intensity: Not reported Tailored: Yes Adherence: mean: 61% (range 0-100%)	*N* = 88 Age = 74.0 (6)	Description: Exercise, nutrition; Exercise (WHO Global Recommendations for Physical Activity for Health for people 60 years and above pamphlet) Nutrition (Canada's Food Guide, and a pedometer)	Complications and length of hospital stay
Ommundsmen, 2018	Country: Norway Cancer type: Colorectal Frailty tool: Vulnerable Elders Survey Setting: Hospital	*N* = 53 Age = 78.2 (7.4)	Description: Multi (Geriatric assessment and Tailored intervention: exercise, nutrition, psychological, medicine ...) Provider: Medical doctor specializing in geriatric medicine Mode of delivery: Face-to-face Number of times: One session of geriatric assessment Duration: 3 weeks Intensity: Not reported Tailored: Yes Adherence: Not reported	*N* = 63 Age = 78.8 (7.8)	Description: Usual care	Complications and length of hospital stay

### Frailty Assessment

Frailty assessment tools included the Groningen Frailty Indicator, Fried Frailty Index, Edmonton Frail Scale, Clinical Frailty Score, and Vulnerable Elders Survey (VES-13).^16,22–24,33^ All the included trials used a different frailty tool. Details of the frailty assessment tools are summarized in Table 2.

**Table 2:** Frailty assessment tools

Tool name	No. studies (no. patients)	No. items	Domains	Scoring	Validation	License required	Website
GFI	57	15	8 Domains Mobility Vision Hearing Nutrition Comorbidity Cognition Psychosocial Physical fitness	Scoring system Each domain is scored and interpreted individually. Item scores Items are scored using yes/no, or sometimes. Scoring direction Lower scores indicate better outcomes	Yes	No (citation needed when used)	https://bmcgeriatr.biomedcentral.com/articles/10.1186/1471-2318-13-86
FFI	110	5	5 Domains Weight loss Exhaustion Physical activity Walk time Grip strength	Scoring system Each domain is scored and interpreted individually. Item scores Each domain is assessed using specific criteria, and individuals are scored based on their performance in each domain. Scoring direction Total score can range from 0 (no frailty criteria met) to 5 (all frailty criteria met). The higher the score, the greater the degree of frailty	Yes	No (citation needed when used)	https://academic.oup.com/biomedgerontology/article/56/3/M146/545770?login=true
CFS	182	9	9 Domains Physical function Mobility Cognition Nutritional status Comorbidities Frailty-related symptoms Social support Medication use Overall health status	Scoring system Single descriptor of a person's level of frailty. Item scores 9-point scale ranging from very fit to terminally ill. Scoring direction Lower scores indicate better outcomes	Yes	No (citation needed when used)	https://www.physio-pedia.com/Clinical_Frailty_Scale
VES-13	116	13	13 Domains Age Health status Stooping, crouching, and kneeling Lifting or carrying objects Reaching or extending arms Writing or handling and grasping small objects Walking 400 m Heavy housework Shopping Managing money Walking across room Doing light housework Bathing or showering	Scoring system Each domain is scored and interpreted individually. Item scores Each domain is assessed using specific criteria, and individuals are scored based on their performance in each domain. Scoring direction A score on the VES-13 ≥3 indicates the patient as vulnerable	Yes	No (citation needed when used)	https://www.rand.org/health-care/projects/acove/survey.html
EFS	1	11	9 Domains Cognition General health status Functional independence Social support Medication use Nutrition Mood Continence Functional performance	Scoring system Each domain is scored and interpreted individually. Item scores Items are scored using yes/no, 0-, 1-, or 2-point scales. Scoring direction Sum up the points for all items to obtain the total EFS score. The total score typically ranges from 0 (Not Frail) to 17 (Severe Frailty), lower scores indicate better outcomes	Yes	Yes	https://www.bgs.org.uk/sites/default/files/content/attachment/2018-07-05/efs.pdf

*CFS* Clinical Frailty Score; *EFS* Edmonton Frail Scale; *FFI* Fried Frailty Index; *GFI* Groningen Frailty Indicator; *VES-13* Vulnerable Elders Survey

### Prehabilitation Intervention

One study implemented exercise as the sole component of the prehabilitation intervention.^16^ Two studies (23, 33) combined exercise and nutrition interventions.^23,33^ Two others utilised multidomain interventions, which encompassed physical, nutritional, and psychological therapy as forms of rehabilitation.^22,24^ Patients received the interventions in various settings, including home, hospital, community, and home and hospital prehabilitation unit.^4,16,22,23,33^

### Quality Assessment

**Fig. 2:**
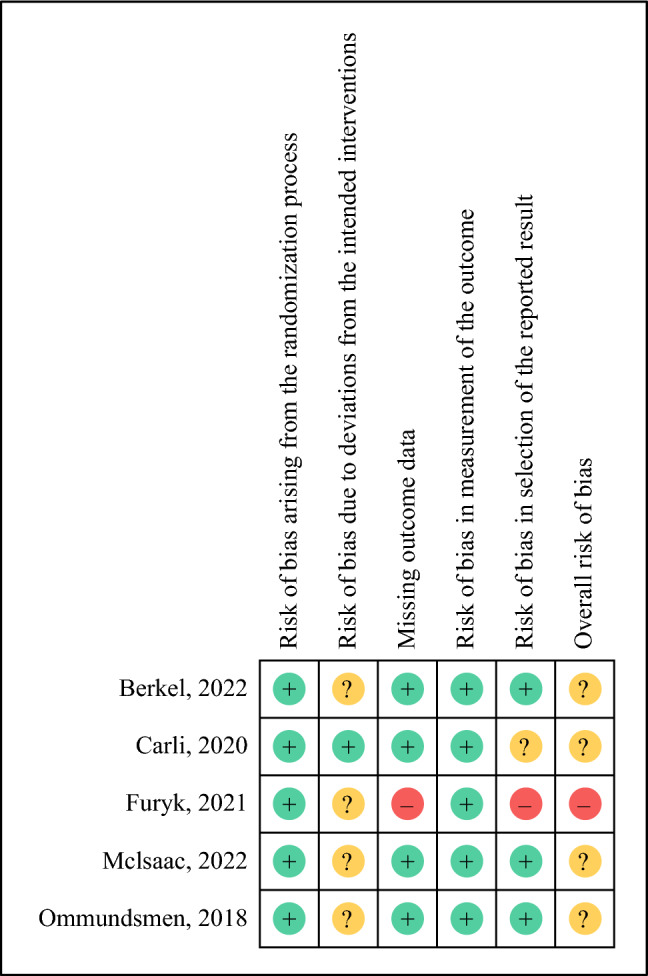
Risk of bias summary

The quality assessment of the included studies is shown in Fig. 2. One study was considered as overall high risk of bias in missing outcome data and selection of the reported result.^33^ Four studies were considered to have some risk of bias.^16,22–24^

### Postoperative Complications

#### Any Complications

**Fig. 3:**
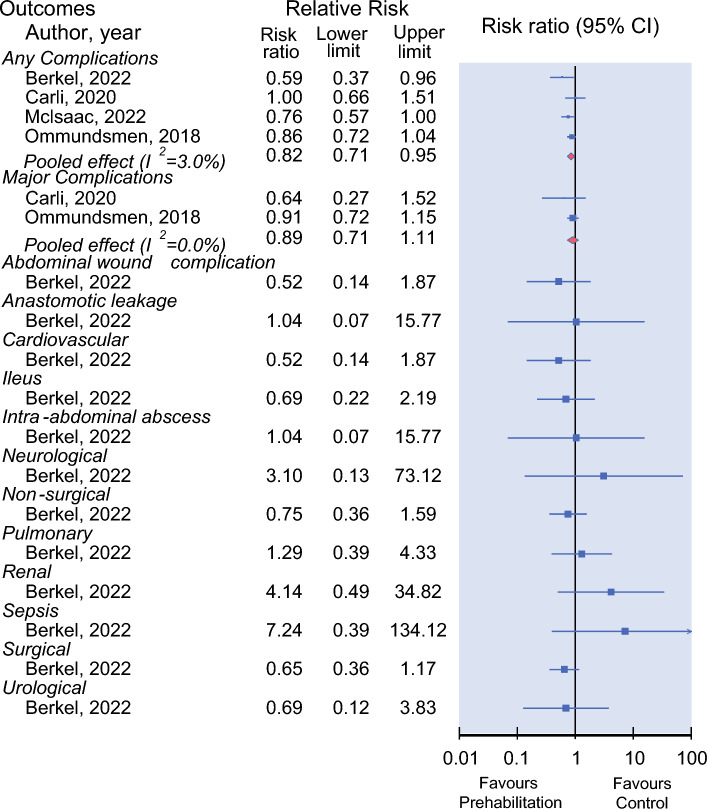
Relative risk for number of postoperative complications in controlled trials on efficacy of prehabilitation for frail patients undergoing cancer surgery. Values <1 favours prehabilitation intervention

Four trials explored the effectiveness of prehabilitation on any complications (including 465 patients).^16,22–24^ The prehabilitation interventions included exercise only, combined exercise and nutrition, and multimodal intervention.^16,22–24^ Prehabilitation was effective in reducing any postoperative complications compared with control (RR = 0.82; 95% CI = 0.71–0.95; Fig. 3). The quality of evidence was rated as high (Table 3).

**Table 3:** Summary of findings and quality of evidence assessment (GRADE)

Outcomes [author, year]	Summary of findings		Quality of evidence assessment (GRADE)				
	Sample (studies)	Effect size (95% CI)	Risk of bias	Inconsistency	Imprecision	Publication bias	Overall quality of evidence
Any complication [Berkel, 2022; Mclsaac, 2022; Ommundsmen, 2018; Carli, 2020]	465 (4 RCTs)	RR: 0.82 (0.71 to 0.95)	Not serious	Not serious	Not serious	Undetected	High
Major complications [Carli, 2020; Ommundsmen, 2018]	226 (2 RCTs)	RR: 0.89 (0.71 to 1.11)	Not serious	Not serious	serious	Undetected	Moderate
Abdominal wound complication [Berkel, 2022]	57 (1 RCT)	RR: 0.52 (0.14 to 1.87)	Not serious	Serious	Serious	Undetected	Low
Anastomotic leakage [Berkel, 2022; Ommundsmen, 2018]	173 (2 RCTs)	RR: 1.04 (0.07 to 15.77)	Not serious	Not serious	Serious	Undetected	Moderate
Cardiovascular [Berkel, 2022]	57 (1 RCT)	RR: 0.52 (0.14 to 1.87)	Not serious	Serious	Serious	Undetected	Low
Ileus [Berkel, 2022]	57 (1 RCT)	RR: 0.69 (0.22 to 2.19)	Not serious	Serious	Serious	Undetected	Low
Intra-abdominal abscess [Berkel, 2022]	57 (1 RCT)	RR: 1.04 (0.07 to 15.77)	Not serious	Serious	Serious	Undetected	Low
Neurological [Berkel, 2022]	57 (1 RCT)	RR: 3.10 (0.13 to 73.12)	Not serious	Serious	Serious	Undetected	Low
Nonsurgical [Berkel, 2022]	57 (1 RCT)	RR 0.75 (0.36 to 1.59)	Not serious	Serious	Serious	Undetected	Low
Pulmonary [Berkel, 2022]	57 (1 RCT)	RR: 1.29 (0.39 to 4.33)	Not serious	Serious	Serious	Undetected	Low
Renal [Berkel, 2022]	57 (1 RCT)	RR: 4.14 (0.49 to 34.82)	Not serious	Serious	Serious	Undetected	Low
Sepsis [Berkel, 2022]	57 (1 RCT)	RR: 7.24 (0.39 to 134.12)	Not serious	Serious	Serious	Undetected	Low
Surgical [Berkel, 2022]	57 (1 RCT)	RR: 0.65 (0.36 to 1.17)	Not serious	Serious	Serious	Undetected	Low
Urological [Berkel, 2022]	57 (1 RCT)	RR: 0.69 (0.12 to 3.83)	Not serious	Serious	Serious	Undetected	Low
Length of hospital stay [Berkel, 2022; Mclsaac, 2022; Carli, 2020]	349 (3 RCTs)	MD: 0.3 (−0.68 to 1.28)	Not serious	Not serious	Serious	Undetected	Moderate

#### Major Complications

Major complications referred to postoperative complications with a CDC grade ≥3. Two trials, including 226 patients, investigated the effectiveness of multimodal interventions on major complications.^22,24^ No effect of prehabilitation on major complications was observed compared with control (RR = 0.89; 95% CI = 0.71–1.11; Fig. 3). The quality of evidence was rated as moderate (Table 3).

#### Other Complications

One trial (including 57 patients) investigated the effect of exercise-only sessions with aerobic and resistance training on a range of postoperative complications.^16^ No difference was observed between the groups, with the quality of evidence rated as low (Table 3). One trial (including 1 patient) investigated the effect of combined exercise and nutrition on a range of postoperative complications, but only five patients were able to be randomised, of which one patient in control group alone completed the entire study to follow-up.^33^ No results of complications and LOS are available; the RoB of this trial was high (Fig. 2).

### Length of Hospital Stay

**Fig. 4:**
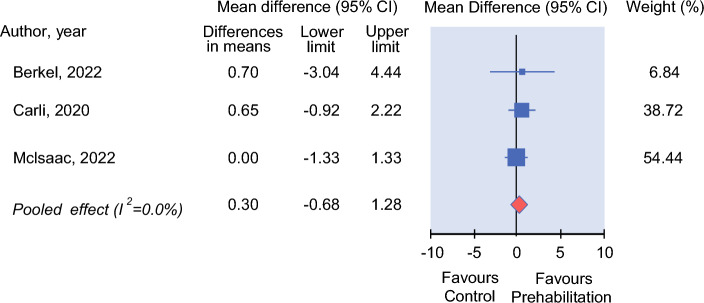
Mean difference for postoperative length of hospital stay (days) in controlled trials on efficacy of prehabilitation for frail patients undergoing cancer surgery. Positive values favours prehabilitation interventions

Three trials (including 349 patients) investigated the effect of prehabilitation on LOS.^16,22,23^ The prehabilitation programs included exercise only, combined exercise and nutrition, and multimodal interventions.^16,22,23^ Pooled estimates demonstrated no significant difference observed in LOS between the prehabilitation and control groups (MD = 0.3; 95% CI = −0.68 to 1.28; Fig. 4). The quality of evidence was rated as moderate (Table 3).

## Discussion

This review identified a small number of randomised controlled trials investigating the effectiveness of prehabilitation in frail patients with cancer undergoing surgery. The frailty tools utilised were different across all included trials. Prehabilitation was effective in reducing the rate of any postoperative complications. However, no difference was observed on other postoperative outcomes, including major postoperative complication and LOS, compared with control.

Some limitations should be considered in this study. The varied prehabilitation interventions and differing definitions of postoperative outcomes across studies introduce significant heterogeneity, complicating the analysis. The prehabilitation interventions reported within the five identified trials include exercise-only sessions (*n* = 57), combined exercise and nutritional interventions (*n* = 183), and the multimodal approach combines exercise, nutrition, and psychological strategies (*n* = 110), or multimodal sessions consisting of geriatric assessment and tailored interventions that cover exercise, nutrition, psychological support, and medical care (*n* = 116).^16,22,23,33^ Additionally, the assessment tools used to measure outcomes, such as the CCI and the CDC, were not uniform across trials, further complicating the analysis. Moreover, the inclusion of a mixed population with different types of gastrointestinal cancers limits the generalizability of the results. Additionally, only three of the five included trials reported on length of stay.^16,22,23^ Two included studies reported on major complications^22,24^; neither found statistically significant differences between prehabilitation and control groups. The small number of trials and sample size reduces the statistical power, affecting the reliability of the findings. Also, among the five included trials, four^16,22,24,33^ focused exclusively on colorectal cancer patients, whereas only one^23^ trial included a broader cancer population (including colorectal, thoracic, hepatobiliary, or urologic cancers). Given this distribution, the findings of our review are primarily applicable to colorectal cancer patients rather than the broader frail cancer population. Future studies should investigate whether similar benefits of prehabilitation extend to other cancer types. These issues highlight the need for more standardized methodologies in future research. The feasibility of conducting a randomised controlled trial in frail patients was highlighted as a significant issue by the study conducted by Furyk et al.^33^ Of the 106 patients initially identified in their trial, only five were randomised, and four of these subsequently dropped out. Notably, approximately 32% of the identified patients (23/106) either declined participation or would not wait to complete the screening. Several barriers that contributed to patients declining participation or not complying with trial processes were identified. These included the physical and psychological effects of frailty, timing of recruitment (patients were busy), and other logistical issues. This should be carefully considered in future trials investigating the effectiveness of prehabilitation in frail patients undergoing cancer surgery. The current evidence is limited by the small number of trials, sample sizes, and to colorectal cancer patients undergoing surgery. To enhance generalizability, future research should include diverse cancer populations beyond colorectal cancer. Additionally, larger randomised controlled trials with adequate power are needed to assess the impact of prehabilitation on length of stay and postoperative complications with greater statistical certainty.

This systematic review and meta-analysis have several strengths. The methodology adheres to the rigorous standards of the *Cochrane Handbook for Systematic Reviews of Interventions*. Our comprehensive search strategy, developed with a librarian from The University of Sydney, included all records up to December 11, 2023, without language limitations, ensuring a thorough evaluation of available literature. We exclusively incorporated the latest RCTs, enhancing the reliability of our findings. The use of validated assessment tools, including RoB2 and GRADE, along with the preregistration of our research protocol, further validates our results. Additionally, employing the comprehensive meta-analysis software for our meta-analyses ensured precise statistical analysis.

Frail patients with cancer undergoing surgery are vulnerable and at higher risk of developing postoperative complications, and longer LOS.^34–38^ Because frailty is an independent predictor for postoperative complications and LOS in older surgical patients and because frailty is modifiable, prehabilitation aimed at enhancing preoperative health and addressing risk factors for frailty has the potential to positively impact long-term postoperative outcomes.^7,10^ In our review, only one included trial reported significant effects of prehabilitation on frail patients with cancer.^16^ Our meta-analysis (including 465 patients) demonstrated the effectiveness of prehabilitation in reducing the rate of any postoperative complications. However, other four included studies did not find any improvements in postoperative outcomes, including postoperative major complications (CDC ≥ 3) and LOS.^22–24,33^

Poor preoperative conditions of patients with cancer and the preoperative period provide a good opportunity for prehabilitation to improve patients’ preoperative status.^39^ Prehabilitation has been validated to improve postoperative outcomes by implementing physical, psychological, and nutritional treatments before cancer surgery.^40,41^ Among the included five trials, the prehabilitation program includes exercise-only sessions with aerobic and resistance training, combined exercise (that incorporates warm-up, strength, core/balance, aerobic exercises, cool-down exercises, and flexibility) and nutritional (providing dietary advice in line with Australian Dietary Guidelines, or provide foundational nutritional advice and cooking tips, and motivate for healthy eating).^16,22–24,33^ Additionally, the multimodal approach combines exercise (aerobic, resistance, and stretching exercises), nutrition (protein supplements), and psychological strategies (personalized coping strategies), or multimodal sessions consisting of geriatric assessment and tailored interventions that cover exercise, nutrition, psychological support, and medical care.^22,24^ The intensity of interventions varied from moderate to moderate-to-high, and the duration ranged from 3 to 4 weeks, with the frequency varying from one to three sessions per week. Thus, differences in the components of the prehabilitation program, as well as the varied intensity, duration, and frequency of interventions, may explain the inconsistent results. This has also been reported as an issue in other systematic reviews of patients with cancer.^26^ Additionally, adherence to the prehabilitation program in the included studies varied from 61 to 80%.^22,23^ Strategies to improve adherence to prehabilitation programs may result in better postoperative outcomes in frail patients with cancer.

Guo et al.^25^ conducted a systematic review and meta-analysis to examine the impact of prehabilitation on postoperative outcomes among frail patients with cancer. The findings revealed that prehabilitation positively influences any and major (CDC ≥ 3) postoperative complications and LOS. They included RCTs and historically controlled trials in their analysis but did not stratify them, potentially leading to lower quality evidence. Chang et al.^26^ conducted a systematic review to explore the effects of prehabilitation on frail colorectal patients with cancer. The study revealed that prehabilitation positively improved major postoperative complications and LOS. However, when the included RCTs and non-RCTs were analysed separately, negative effects of prehabilitation on postoperative complications (CDC ≥ 3) and LOS were found in the meta-analysis of included RCTs, but positive effects were found in non-RCTs. Thus, the methodological issue could explain why their findings differ from ours. Furthermore, Guo et al.^25^ suggested that a multimodal prehabilitation program, with exercise as a central component, may be especially beneficial for these patients. In our review, all included trials incorporated exercise into their prehabilitation programs.^16,22–24,33^ However, only one trial demonstrated significant effects of prehabilitation on postoperative complications and LOS.^16^ This trial was unique in conducting exercise at moderate-to-high intensity with three sessions per-week, whereas the others utilized only moderate intensity or lower intervention times. This variation may account for the inconsistent results. Seynnes et al.^42^ reported that high-intensity resistance exercises significantly increased muscle strength in frail, older individuals. Similarly, Sahin et al.^43^ found that high-intensity exercise considerably improved physical performance compared with low-intensity exercise in the frail, older patients. These findings suggest that researchers should consider intensifying the exercise component in prehabilitation programs to further explore its effects on frail patients with cancer.

In our systematic review, the selected articles utilised various validated tools to screen for frailty in patients with cancer. These tools include the Clinical Frailty Score, Edmonton Frail Scale, Fried Frailty Index, Groningen Frailty Indicator, and the Vulnerable Elders Survey (VES-13).^16,22–24,33^ Each of these instruments has been proven effective for assessing frailty, highlighting their reliability and widespread adoption in clinical research. However, these tools differ in their approaches and criteria, which may introduce variability in identifying frail patients. This variability could potentially lead to biases in patient selection across studies, consequently affecting the comparability of outcomes. It is crucial to consider these differences when interpreting the results of this review, as they may contribute to the observed discrepancies in research findings.

## Conclusions

This systematic review and meta-analysis aimed to examine the effectiveness of prehabilitation on frail patients with cancer undergoing surgery. Our findings suggest that prehabilitation may reduce the rate of any postoperative complications in this vulnerable group. However, the effect of prehabilitation, when compared to control, on other postoperative outcomes is still unclear due to the small number of trials available in the current literature. Additional large-scale, high-quality trials are needed to determine the definitive role of prehabilitation in frail patients undergoing cancer surgery.

## Supplementary Information

Below is the link to the electronic supplementary material.

Supplementary file1 (DOCX 17 kb)

## References

[1] Organization WH. 2024. https://www.who.int/news/item/01-02-2024-global-cancer-burden-growing--amidst-mounting-need-for-services on 28 May 2024 News release.

[2] Lordick F, Carneiro F, Cascinu S, Fleitas T, Haustermans K, Piessen G, et al. Gastric cancer: ESMO clinical practice guideline for diagnosis, treatment and follow-up. *Ann Oncol*. 2022;33(10):1005–20. 35914639 10.1016/j.annonc.2022.07.004

[3] Ethun CG, Bilen MA, Jani AB, Maithel SK, Ogan K, Master VA. Frailty and cancer: implications for oncology surgery, medical oncology, and radiation oncology. *CA Cancer J Clin*. 2017;67(5):362–77. 28731537 10.3322/caac.21406

[4] Ommundsen N, Nesbakken A, Wyller TB, Skovlund E, Bakka AO, Jordhoy MS, et al. Post-discharge complications in frail older patients after surgery for colorectal cancer. *Eur J Surg Oncol*. 2018;44(10):1542–7. 30037638 10.1016/j.ejso.2018.06.024

[5] Hegazi RA, Hustead DS, Evans DC. Preoperative standard oral nutrition supplements vs immunonutrition: results of a systematic review and meta-analysis. *J Am Coll Surg*. 2014;219(5):1078–87. 25260681 10.1016/j.jamcollsurg.2014.06.016

[6] Gillis C, Ljungqvist O, Carli F. Prehabilitation, enhanced recovery after surgery, or both? A narrative review. *Br J Anaesth*. 2022;128(3):434–48. 35012741 10.1016/j.bja.2021.12.007

[7] Makary MA, Segev DL, Pronovost PJ, Syin D, Bandeen-Roche K, Patel P, et al. Frailty as a predictor of surgical outcomes in older patients. *J Am Coll Surg*. 2010;210(6):901–8. 20510798 10.1016/j.jamcollsurg.2010.01.028

[8] Arya S, Kim SI, Duwayri Y, Brewster LP, Veeraswamy R, Salam A, et al. Frailty increases the risk of 30-day mortality, morbidity, and failure to rescue after elective abdominal aortic aneurysm repair independent of age and comorbidities. *J Vasc Surg*. 2015;61(2):324–31. 25312534 10.1016/j.jvs.2014.08.115

[9] van de Ree CLP, Landers MJF, Kruithof N, de Munter L, Slaets JPJ, Gosens T, et al. Effect of frailty on quality of life in elderly patients after hip fracture: a longitudinal study. *BMJ Open*. 2019;9(7):e025941. 31324679 10.1136/bmjopen-2018-025941 PMC6661564

[10] Dent E, Martin FC, Bergman H, Woo J, Romero-Ortuno R, Walston JD. Management of frailty: opportunities, challenges, and future directions. *Lancet*. 2019;394(10206):1376–86. 31609229 10.1016/S0140-6736(19)31785-4

[11] Beggs T, Sepehri A, Szwajcer A, Tangri N, Arora RC. Frailty and perioperative outcomes: a narrative review. *Can J Anaesth*. 2015;62(2):143–57. 25420470 10.1007/s12630-014-0273-z

[12] McIsaac DI, Moloo H, Bryson GL, van Walraven C. The association of frailty with outcomes and resource use after emergency general surgery: a population-based cohort study. *Anesth Analg*. 2017;124(5):1653–61. 28431425 10.1213/ANE.0000000000001960

[13] Tepas JJ 3rd. Simple frailty score predicts postoperative complications across surgical specialties. *Am J Surg*. 2013;206(5):818. 24119893 10.1016/j.amjsurg.2013.07.029

[14] Kow AW. Prehabilitation and its role in geriatric surgery. *Ann Acad Med Singap*. 2019;48(11):386–92. 31960020

[15] Silver JK, Baima J, Mayer RS. Impairment-driven cancer rehabilitation: an essential component of quality care and survivorship. *CA Cancer J Clin*. 2013;63(5):295–317. 23856764 10.3322/caac.21186

[16] Berkel AEM, Bongers BC, Kotte H, Weltevreden P, de Jongh FHC, Eijsvogel MMM, et al. Effects of community-based exercise prehabilitation for patients scheduled for colorectal surgery with high risk for postoperative complications: results of a randomized clinical trial. *Ann Surg*. 2022;275(2):e299-306. 33443905 10.1097/SLA.0000000000004702 PMC8746915

[17] Colman RW. Humoral mediators of catastrophic reactions associated with protamine neutralization. *Anesthesiology*. 1987;66(5):595–6. 3578874

[18] Michael CM, Lehrer EJ, Schmitz KH, Zaorsky NG. Prehabilitation exercise therapy for cancer: a systematic review and meta-analysis. *Cancer Med*. 2021;10(13):4195–205. 34110101 10.1002/cam4.4021 PMC8267161

[19] Minnella EM, Awasthi R, Gillis C, Fiore JF Jr, Liberman AS, Charlebois P, et al. Patients with poor baseline walking capacity are most likely to improve their functional status with multimodal prehabilitation. *Surgery*. 2016;160(4):1070–9. 27476586 10.1016/j.surg.2016.05.036

[20] Silver JK. Cancer rehabilitation and prehabilitation may reduce disability and early retirement. *Cancer*. 2014;120(14):2072–6. 24752917 10.1002/cncr.28713

[21] Silver JK. Cancer prehabilitation and its role in improving health outcomes and reducing health care costs. *Semin Oncol Nurs*. 2015;31(1):13–30. 25636392 10.1016/j.soncn.2014.11.003

[22] Carli F, Bousquet-Dion G, Awasthi R, Elsherbini N, Liberman S, Boutros M, et al. Effect of multimodal prehabilitation vs postoperative rehabilitation on 30-day postoperative complications for frail patients undergoing resection of colorectal cancer: a randomized clinical trial. *JAMA Surg*. 2020;155(3):233–42. 31968063 10.1001/jamasurg.2019.5474 PMC6990653

[23] McIsaac DI, Hladkowicz E, Bryson GL, Forster AJ, Gagne S, Huang A, et al. Home-based prehabilitation with exercise to improve postoperative recovery for older adults with frailty having cancer surgery: the PREHAB randomised clinical trial. *Br J Anaesth*. 2022;129(1):41–8. 35589429 10.1016/j.bja.2022.04.006

[24] Ommundsen N, Wyller TB, Nesbakken A, Bakka AO, Jordhoy MS, Skovlund E, et al. Preoperative geriatric assessment and tailored interventions in frail older patients with colorectal cancer: a randomized controlled trial. *Colorectal Dis*. 2018;20(1):16–25. 28649755 10.1111/codi.13785

[25] Guo Y, Ding L, Miao X, Jiang X, Xu T, Xu X, et al. Effects of prehabilitation on postoperative outcomes in frail cancer patients undergoing elective surgery: a systematic review and meta-analysis. *Support Care Cancer*. 2022;31(1):57. 36534300 10.1007/s00520-022-07541-1

[26] Chang MC, Choo YJ, Kim S. Effect of prehabilitation on patients with frailty undergoing colorectal cancer surgery: a systematic review and meta-analysis. *Ann Surg Treat Res*. 2023;104(6):313–24. 37337603 10.4174/astr.2023.104.6.313 PMC10277181

[27] Higgins JPT TJ, Chandler J, Cumpston M, Li T, Page MJ, Welch VA (editors)**.** Cochrane handbook for systematic reviews of interventions version 6.4 (updated August 2023). Cochrane. 2023.

[28] Moher D, Liberati A, Tetzlaff J, Altman DG, Group P. Preferred reporting items for systematic reviews and meta-analyses: the PRISMA statement. *PLoS Med*. 2009;6(7):e1000097. 19621072 10.1371/journal.pmed.1000097 PMC2707599

[29] Bai Z, Hirst N, Koh C, Shahab R, Steffens D. 2024. https://osf.io/2m8bf. Accessed 10 May 2024.

[30] Accessed at Veritas Health Innovation at www.covidence.org.

[31] Higgins JP, Green S. Cochrane handbook for systematic reviews of interventions. 2008.

[32] Guyatt GH, Oxman AD, Vist GE, Kunz R, Falck-Ytter Y, Alonso-Coello P, et al. GRADE: an emerging consensus on rating quality of evidence and strength of recommendations. *BMJ*. 2008;336(7650):924–6. 18436948 10.1136/bmj.39489.470347.AD PMC2335261

[33] Furyk C, Senthuran S, Nye D, Ho YH, Leicht AS. Prehabilitation for frail patients undergoing colorectal surgery: lessons learnt from a randomised feasibility study. *Front Rehabil Sci*. 2021;2:650835. 36188831 10.3389/fresc.2021.650835 PMC9397917

[34] de Arruda FN, Oonk MHM, Mourits MJE, de Graeff P, Jalving M, de Bock GH. Determinants of health-related quality of life in elderly ovarian cancer patients: the role of frailty and dependence. *Gynecol Oncol*. 2019;153(3):610–5. 30935716 10.1016/j.ygyno.2019.03.249

[35] Kirkhus L, Saltyte Benth J, Gronberg BH, Hjermstad MJ, Rostoft S, Harneshaug M, et al. Frailty identified by geriatric assessment is associated with poor functioning, high symptom burden and increased risk of physical decline in older cancer patients: prospective observational study. *Palliat Med*. 2019;33(3):312–22. 30712456 10.1177/0269216319825972 PMC6376598

[36] Vermeiren S, Vella-Azzopardi R, Beckwee D, Habbig AK, Scafoglieri A, Jansen B, et al. Frailty and the prediction of negative health outcomes: a meta-analysis. *J Am Med Dir Assoc*. 2016;17(12):1163 e1-1163 e17. 27886869 10.1016/j.jamda.2016.09.010

[37] Michaud Maturana M, English WJ, Nandakumar M, Li Chen J, Dvorkin L. The impact of frailty on clinical outcomes in colorectal cancer surgery: a systematic literature review. *ANZ J Surg*. 2021;91(11):2322–9. 34013571 10.1111/ans.16941

[38] Meyers BM, Al-Shamsi HO, Rask S, Yelamanchili R, Phillips CM, Papaioannou A, et al. Utility of the Edmonton Frail Scale in identifying frail elderly patients during treatment of colorectal cancer. *J Gastrointest Oncol*. 2017;8(1):32–8. 28280606 10.21037/jgo.2016.11.12 PMC5334059

[39] van der Hulst HC, Bastiaannet E, Portielje JEA, van der Bol JM, Dekker JWT. Can physical prehabilitation prevent complications after colorectal cancer surgery in frail older patients? *Eur J Surg Oncol*. 2021;47(11):2830–40. 34127328 10.1016/j.ejso.2021.05.044

[40] Molenaar CJ, van Rooijen SJ, Fokkenrood HJ, Roumen RM, Janssen L, Slooter GD. Prehabilitation versus no prehabilitation to improve functional capacity, reduce postoperative complications and improve quality of life in colorectal cancer surgery. *Cochrane Database Syst Rev*. 2022;5(5):CD013259. 35588252 10.1002/14651858.CD013259.pub2 PMC9118366

[41] Coderre D, Brahmbhatt P, Hunter TL, Baima J. Cancer prehabilitation in practice: the current evidence. *Curr Oncol Rep*. 2022;24(11):1569–77. 35788874 10.1007/s11912-022-01304-1

[42] Seynnes O, Fiatarone Singh MA, Hue O, Pras P, Legros P, Bernard PL. Physiological and functional responses to low-moderate versus high-intensity progressive resistance training in frail elders. *J Gerontol A Biol Sci Med Sci*. 2004;59(5):503–9. 15123761 10.1093/gerona/59.5.m503

[43] Sahin UK, Kirdi N, Bozoglu E, Meric A, Buyukturan G, Ozturk A, et al. Effect of low-intensity versus high-intensity resistance training on the functioning of the institutionalized frail elderly. *Int J Rehabil Res*. 2018;41(3):211–7. 29620558 10.1097/MRR.0000000000000285

