# Supplementary material for: Prehabilitation in Frail Patients Undergoing Cancer Surgery: A Systematic Review and Meta-analysis
Source: Ann Surg Oncol. 2025 Jun 4;32(10):7707–22. doi: 10.1245/s10434-025-17589-y (PMC12454461; doi:10.1245/s10434-025-17589-y)
Supplement: Supplementary file 1 — Supplementary file1 (DOCX 17 kb) [file 10434_2025_17589_MOESM1_ESM.docx]

**Supplementary Table S1. Search strategy in Medline**

1 exp Randomized Controlled Trials as Topic/

2 randomi?ed controlled trial.mp.

3 exp Controlled Clinical Trial/

4 controlled clinical trial.mp.

5 randomi?ed.tw.

6 clinical trial.tw.

7 trial.tw.

8 quasi-randomi?ed.tw.

9 placebo.tw.

10 random$.tw.

11 RCT.tw.

12 1 or 2 or 3 or 4 or 5 or 6 or 7 or 8 or 9 or 10 or 11

13 exp Preoperative Period/

14 pre?operat$.mp.

15 before surgery.mp.

16 before operation.mp.

17 exp Preoperative Care/

18 13 or 14 or 15 or 16 or 17

19 exp Neoplasms/

20 neoplasm$.mp.

21 cancer.mp.

22 tumo?r$.mp.

23 malignan$.mp.

24 19 or 20 or 21 or 22 or 23

25 exp Postoperative Complications/

26 post?operat$ complication$.mp.

27 adverse effect$.mp.

28 adverse event$.mp.

29 Clavien-Dindo.mp.

30 exp Morbidity/

31 morbidit$.mp.

32 length of hospital stay.mp.

33 exp "Length of Stay"/

34 length of stay$.mp.

35 hospital stay$.mp.

36 LOS.tw.

37 25 or 26 or 27 or 28 or 29 or 30 or 31 or 32 or 33 or 34 or 35 or 36

38 exp Exercise/

39 exercis$.mp.

40 exp Physical Fitness/

41 physical fitnes$.mp.

42 exp Exercise Therapy/

43 exercis$ therap$.mp.

44 physical activit$.mp.

45 exp Rehabilitation/

46 prehabilitation.mp.

47 rehabilit$.mp.

48 exercise train$.mp.

49 physical therap$.mp.

50 38 or 39 or 40 or 41 or 42 or 43 or 44 or 45 or 46 or 47 or 48 or 49

51 exp diet/

52 diet$.mp.

53 nutrition$.mp.

54 diet$ counselling.mp.

55 exp Enteral Nutrition/

56 enteral nutrition$.mp.

57 exp diet therapy/

58 nutrition$ therap$.mp.

59 exp parenteral nutrition/

60 exp nutritional support/

61 nutrition$ support.mp.

62 exp food/

63 51 or 52 or 53 or 54 or 55 or 56 or 57 or 58 or 59 or 60 or 61 or 62

64 exp cognitive behavioral therapy/

65 cognitive behavio?ral therap$.mp.

66 Relaxation.mp.

67 exp mindfulness/

68 mindfulness.mp.

69 exp Adaptation, Psychological/

70 coping.mp.

71 psychosocial intervention$.mp.

72 exp psychotherapy/

73 psychotherap$.mp.

74 CBT.tw.

75 exp mental health/

76 Psychological.mp.

77 psychoeducation.mp.

78 64 or 65 or 66 or 67 or 68 or 69 or 70 or 71 or 72 or 73 or 74 or 75 or 76 or 77

79 50 or 63 or 78

80 12 and 18 and 24 and 37 and 79
